# Effects of various beverages on characteristics of provisional restoration materials

**DOI:** 10.1002/cre2.842

**Published:** 2024-04-10

**Authors:** Praween Wayakanon, Teeraphan Narakaew, Kornchanok Wayakanon

**Affiliations:** ^1^ Department of Oral Biology, Faculty of Dentistry Naresuan University Phitsanulok Thailand; ^2^ Department of Restorative Dentistry, Faculty of Dentistry Naresuan University Phitsanulok Thailand

**Keywords:** beverages, color, hardness and roughness, provisional materials

## Abstract

**Objective:**

To investigate the effect of common beverages on four currently used provisional restoration materials: Protemp®4, Integrity®, polymethyl methacrylate (PMMA) block, and acrylic resin. Flowable resin composite is included as a control group.

**Materials and Methods:**

Each material was formed into disks of 10‐mm diameter and 4‐mm thickness (*N* = 40) by loading the material into acrylic molds. The exposed surface in the mold was covered using a glass slide to prevent an oxygen inhibition layer, and polymerization then proceeded. The solidified disks were placed in distilled water for 24 h. These samples (*n* = 8) were then immersed for 14 days in one of four different beverages: water, orange juice, cola, and coffee. Changes in color dimension, hardness, and roughness were observed and then analyzed using two‐way repeated analysis of variance.

**Results:**

The provisional materials had more obvious changes in all three color dimensions than the flowable resin composite. Integrity showed the biggest changes, followed by acrylic resin and PMMA block, whereas Protemp had the smallest changes. The hardness of all the materials significantly decreased after immersion in any of the beverages for 14 days. There were no changes in surface roughness when the materials were immersed in distilled water. The surface roughness of the PMMA block significantly decreased in orange juice whereas that of Integrity and acrylic resin significantly increased in cola.

**Conclusion:**

Different kinds of provisional materials had different degrees of staining due to their composition. Moisture had a significant influence on the hardness of materials, and the acidity of cola significantly roughened the surface of the provisional materials.

## 1 INTRODUCTION

A provisional or interim restoration is designed to protect dental pulp tissue and periodontium, preventing displacement of the treated tooth and maintaining its functionality for a limited period of time, while waiting for the permanent restoration. Several types of materials can be used for a provisional restoration, with the most common being polymethyl methacrylate (PMMA, C_5_O_2_H_8_) and bis‐acryl resin (Burns et al., 2003).

There are various types of PMMA, differing in their polymerization procedure: heat‐, chemical‐, microwave‐, or light‐cured PMMA. Two of these are common choices for provisional restorations. Chemical‐cured PMMA is frequently used when the provisional restoration is built chairside, that is, directly in the mouth. The chemical polymerization initiator, tertiary amine, activates benzoyl peroxide, resulting in the generation of free radicals that initiate polymerization, but also resulting in a substantial amount of heat (Manak & Arora, 2011). This causes the pulpal temperature to typically rise 0.42°C–7.21°C above normal. If the pulpal temperature increases by 5.6°C, 15% of cases can experience some amount of pulp necrosis. A pulpal temperature rise of 11.2°C or 16.8°C can cause pulp necrosis in 60% or 100% pulp of cases, respectively (Lipski et al., 2020). The degree of polymerization of the chemical‐cured PMMA, only 60%, is markedly lower than that of the heat‐cured version, which is 95% (Bayraktar et al., 2006). Consequently, the uncured residual monomers will leach out, which can be harmful to pulpal tissue and periodontium. They can also dissolve into the surrounding saliva (Ansteinsson et al., 2013). Compared to the others, the chemical‐cured PMMA also has high shrinkage during polymerization, approximately 6.15%–8.7% (Kwon et al., 2014), as well as voids in the material and low color stability (Schwantz et al., 2017).

A second type of PMMA that is commonly chosen for provisional restorations is used with computer‐assisted design/computer‐assisted machining (CAD‐CAM), that is, outside the mouth. This solid, bulk PMMA used in CAD‐CAM is polymerized commercially from acrylic resin using both high temperature and high pressure. This process results in a material with less porosity than the chairside chemical‐cured PMMA and with superior mechanical properties as well (Kelvin Khng et al., 2016).

Unlike the two types of PMMA described, which are acrylic resin based, bis‐acryl resin composite is a hydrophobic dimethacrylate material (Saisadan et al., 2016). Its properties surpass acrylic resin in many ways: less shrinkage and heat when setting, odorless, stronger, more esthetic, and better water resistance (Heboyan et al., 2019; Saisadan et al., 2016). However, studies on its color stability vary in their results. Specifically, the glass filler particles in its composition cause the bis‐acryl resin composite to have significantly lower shrinkage than acrylic resin (1%–1.7% vs. 6%, respectively), providing better marginal adaptation. Although bis‐acryl resin composite has low flexural strength in the first 30 min after fabrication due to relatively slow initial polymerization, the strength increases greatly after 24 h (Balkenhol et al., 2007, 2008).

Almost all provisional materials for dental restorations are resin‐based. The staining susceptibility of these materials is related to the types of resin matrix, fillers, and staining agents (Bagheri et al., 2005; Canay & Çehreli, 2003). Liquid absorption mainly occurs in the resin matrix while the glass fillers absorb fluid onto the surface. Therefore, the amount of liquid absorption depends on the resin content and the quality of the bond between the resin and fillers. Excessive water absorption decreases the life of resin‐based materials by expanding the resin component and hydrolyzing silane, causing microcrack formation (Bagheri et al., 2005). These microcracks often occur at the filler–resin matrix interface, resulting in stain penetration and discoloration (Bagheri et al., 2005).

Whichever material is ultimately selected, the patient will use the provisional restoration constantly in daily life while waiting for the permanent restoration, during which time the material will be exposed to many kinds of food and drink for as long as it remains in use. This continual flow of food and drink has the potential to alter the characteristics of the provisional restoration materials, and these changes could in turn affect patient satisfaction, particularly when the front teeth are involved. The purpose of this study is to evaluate changes in the color, hardness, and surface roughness of modern provisional restoration materials when they are in contact with various common beverages.

## 2 MATERIALS AND METHODS

### 2.1 Sample preparation

The four types of provisional restoration materials (*n* = 8) investigated in this study are shown in Table 1, along with their compositions. There are two bis‐acryl resin composites (referred to here as “Protemp” and “Integrity”) and two PMMAs (“PMMA Block” and “Acrylic”). Flowable resin composite (“Flowable”) is included as a control. All of these are shade A2, except for Acrylic, which is ivory shade. Each material was formed into disks of 10‐mm diameter and 4‐mm thickness using acrylic molds, except for the PMMA block, which was formed using CAD‐CAM.

**Table 1:** The four provisional restoration materials and one control investigated in this study, along with their compositions.

Material	Composition
Bis‐acryl resin composite Protemp®4 (3M ESPE) “Protemp”	*Base paste*: Ethoxylatedbisphenol A dimethacrylate (Bis‐EMA) Silane‐treated amorphous silica Silane‐treated silica Reaction products of 1,6‐diisocyanatohexane with 2‐[(2‐methacryloyl) ethyl] 6‐hydroxyhexanoate and 2‐hydroxyethyl mathacrylate (DESMA) *Catalyst paste*: Ethanol, 2,2′‐[(1‐methylethylidene) bis(4,1‐phenyleneoxy)] bis‐, diacetate Benzyl‐phenyl‐barbituric acid Silane‐treated silica Tert‐butyl peroxy‐3,5,5‐trimethylhexanoate
Bis‐acryl resin composite Integrity® (Dentsply Detrey GmbH) “Integrity”	Ethoxylatedbisphenol A dimethacrylate (Bis‐EMA) Methacrylate monomer Barium boron alumino silicate glass Hydrophobic amorphous fumed silica Catalyst Stabilizer
CAD‐CAM PMMA (PMMA Block) T‐140P14 (TD Dental Supply) “PMMA Block”	Proprietary
Chemical‐cured PMMA (acrylic resin) Unifast Trad® (GC Corporation) “Acrylic”	*Powder*: Methyl methacrylate Ethyl methacrylate copolymer *Liquid*: Methyl methacrylate Butylated hydroxytoluene Hydroquinone
Flowable resin composite Filtek™ Supreme Ultra (3M ESPE) “Flowable”	Bisphenol A‐glycidyl methacrylate (Bis‐GMA) Triethylene glycol dimethacrylate (TEGDMA) Procrylate resins Silane‐coated ytterbium trifluoride filler Silane‐coated silica fillers Silane‐coated zirconia/silica cluster filler

A glass slide was placed on top of the Protemp, Integrity, and Acrylic during chemical polymerization for 2 min in the mold. The flowable resin composite was placed in the mold in two layers of 2‐mm thickness, each polymerized for 40 s (Mini LED™ Standard; ACTEON). The top layer was covered using a glass slide during polymerization to prevent an oxygen inhibition layer. The CAD‐CAM PMMA disks were designed using CAD software (Powershape 2020, Autodesk Inc.) and milled using a milling unit (Coritec 250i; imes‐icore GmbH). After the samples were formed, they were stored in distilled water at 37°C for 24 h. Finally, both sides of each sample were polished progressively using sandpaper numbers 600, 800, and 1000 in that order.

### 2.2 Investigation of material characteristics in different beverages

After 24 h in distilled water, each sample was measured for color, hardness, and surface roughness as pretreatment information. The three dimensions (L*a*b*) of color were measured using a spectrophotometer (VITA Easyshade® V; VITA Zahnfabrik) according to International Commission on Illumination (CIE) protocols. The hardness test (ZHVμ Micro Hardness Tester; ZwickRoell Indentec Hardness Testing Machines Limited) was performed with a loading force of 50 g and a dwelling time of 10 s. The surface roughness was analyzed using an Atomic Force Microscope (AFM) (Flex‐Axiom; Nanosurf). The AFM was set to a resonance frequency of 190 kHz and a surface area of 50 × 50 µm^2^. The probe (Tab190AI‐G; Budgetsensors) had a spring constant of 48 N/m.

Eight samples of each kind of material were then immersed in a different beverage (distilled water, orange juice, cola, or coffee, as per Table 2) for 2 weeks with mechanical agitation in a closed chamber at 37°C (Incubating rocking shaker ISRK04HDG; VWR International Company). The beverage itself was replaced once a day to keep it relatively fresh. The samples were washed with distilled water before measuring their color dimensions, hardness, and surface roughness at Days 5, 7, and 14.

**Table 2:** The ingredients of each kind of beverage in this study.

Beverage	pH	Ingredients
Distilled water	7.04	NA
Orange juice (UHT)	3.71	100% Orange juice
Cola (sugar‐free)	2.55	Carbonic acid anhydride, phosphoric acid, trisodium citrate, sucralose, acesulfame potassium
Coffee (canned)	6.20	2% Coffee, 3% sugar, 95% water

Abbreviations: NA, not applicable; UHT, ultra‐high temperature.

### 2.3 Data analysis

The sample size was calculated using G*Power software with the power level set at 80% and the effect size set at 0.5. After the means and standard deviations of the color dimensions were calculated, data analysis showed that the three‐color dimensions were normally distributed according to the Shapiro–Wilk test (*p* = .827). They were analyzed using two‐way repeated analysis of variance (ANOVA), including the Bonferroni post hoc multiple comparison test at the significance level of 0.05. Comparison of hardness was performed using two‐way ANOVA, including the pairwise comparison post hoc test at the significance level of 0.05. All analysis was performed using SPSS statistical software (SPSS 23.0; SPSS Inc.).

## 3 RESULTS

### 3.1 The color dimensions of the provisional restoration materials under different beverages

After the eight samples of each material (Protemp, Integrity, PMMA Block, Acrylic, and Flowable) were immersed in each kind of beverage for 5, 7, and 14 days, their three‐color dimensions (L*a*b*) were examined.

The lightness of the flowable resin composite significantly decreased (*p* < .05) after 5 days in distilled water or coffee. There was no significant change in cola or orange juice throughout the 14 days (*p* > .05). The lightness of Protemp in different beverages was similar to that in distilled water. The lightness decreased significantly (*p* < .05) from Day 5 when immersed in distilled water, orange juice, or coffee, while it significantly decreased in cola on Day 14 (*p* = .006). The lightness of integrity, PMMA block, and acrylic resin significantly decreased (*p* < .05) from Day 5 in all beverages (Table 3).

**Table 3:** The lightness, redness, and yellowness of provisional restoration materials in different beverages for 0–14 days.

	L*				a*				b*			
Beverage and Materials	0 days	5 days	7 days	14 days	0 days	5 days	7 days	14 days	0 days	5 days	7 days	14 days
Water												
Flowable	100 ± 1.18^aA^	98.59 ± 0.20^bA^	99.40 ± 1.16^bA^	97.88 ± 0.37^bA^	100 ± 0.28^aA^	102 ± 0.05^aA^	104.54 ± 0.22^aA^	95.45 ± 0.11^bA^	100 ± 1.43^aA^	97 ± 0.19^aA^	94 ± 1.39^aA^	93.40 ± 0.30^aA^
Protemp	100 ± 0.47^aA^	98.34 ± 1.22^bA^	99.05 ± 0.89^bA^	98.37 ± 1.13^bA^	100 ± 0.23^aA^	89.31 ± 0.30^bB^	91.22 ± 0.25^bA^	87.02 ± 0.25^bA^	100 ± 0.85^aA^	97.46 ± 1.04^aA^	99.11 ± 0.86^aA^	99.48 ± 1.09^aA^
Integrity	100 ± 0.50^aA^	98.15 ± 0.48^bA^	98.40 ± 0.52^bA^	97.63 ± 0.57^bA^	100 ± 0.18^aA^	173.68 ± 0.12^bC^	176.31 ± 0.11^bB^	205.26 ± 0.14^bB^	100 ± 0.54^aA^	99.62 ± 0.4^aA^	101.67 ± 0.42^aA^	101.05 ± 0.55^aA^
PMMA block	100 ± 0.10^aA^	98.73 ± 0.05^bA^	98.84 ± 0.09^bA^	98.23 ± 0.47^bA^	100 ± 0.01^aA^	125 ± 0.05^bA^	140 ± 0.05^bB^	200 ± 0.02^bB^	100 ± 0.17^aA^	98.92 ± 0.17^aA^	100.29 ± 0.24^aA^	101.46 ± 0.21^aA^
Acrylic resin	100 ± 0.35^aA^	98.68 ± 0.39^bA^	99.23 ± 0.34^bA^	98.24 ± 0.53^bA^	100 ± 0.07^aA^	120 ± 0.09^bA^	160 ± 0.07^bB^	180 ± 0.13^bB^	100 ± 0.83^aA^	101.15 ± 0.85^aA^	101.83 ± 0.70^aA^	102.52 ± 0.95^aA^
Orange juice												
Flowable	100 ± 0.85^aA^	99.15 ± 1.22^aA^	99.61 ± 0.81^aA^	99.41 ± 0.95 ^aA^	100 ± 0.21^aA^	106 ± 0.81^aA^	108 ± 0.87^aA^	105 ± 1.15^aA^	100 ± 0.93^aA^	112.77 ± 1.86^bA^	113.57 ± 1.97^bA^	120.32 ± 5.50^bA^
Protemp	100 ± 0.34^aA^	99.20 ± 0.47^bA^	99.33 ± 0.54^bA^	99.23 ± 0.53^bA^	100 ± 0.17^aA^	106.45 ± 0.01^aA^	107.66 ± 0.15^aA^	101.20 ± 0.13^aA^	100 ± 1.09^aA^	103.5 ± 0.74^aA^	105.46 ± 1.60^aA^	105.90 ± 2.71^aA^
Integrity	100 ± 0.50^aA^	98.86 ± 0.63^bA^	98.44 ± 0.59^bA^	98.66 ± 0.48^bA^	100 ± 0.22^aA^	184.61 ± 0.23^bB^	184.61 ± 0.23^bB^	202.56 ± 0.19^bB^	100 ± 0.69^aA^	111.84 ± 1.63^bA^	114.25 ± 2.18^bA^	116.05 ± 2.83^bA^
PMMA block	100 ± 0.10^aA^	98.46 ± 0.22^bA^	98.75 ± 0.13^bA^	98.65 ± 0.18^bA^	100 ± 0.01^aA^	100 ± 0.01^bA^	120 ± 0.03^bA^	150 ± 0.05^bA^	100 ± 0.16^aA^	101.77 ± 0.16^bA^	102.61 ± 0.21^bA^	103.60 ± 0.31^bA^
Acrylic resin	100 ± 0.67^aA^	99.38 ± 0.48^bA^	99.38 ± 0.66^bA^	99.32 ± 0.55^bA^	100 ± 0.11^aA^	95 ± 0.08^bA^	85 ± 0.15^bA^	62 ± 0.03^bA^	100 ± 1.23^aA^	104.48 ± 0.86^bA^	105.15 ± 0.84^bA^	106.80 ± 1.03^bA^
Cola												
Flowable	100 ± 0.97^aA^	99.88 ± 1.11^aA^	99.15 ± 1.01^aA^	99.98 ± 1.10^aA^	100 ± 0.23^aA^	102.70 ± 0.34^aA^	106 ± 0.36^aA^	108 ± 0.26^aA^	100 ± 1.50^aA^	98 ± 1.96^aA^	98.75 ± 1.42^aA^	99.54 ± 1.64^aA^
Protemp	100 ± 0.26^aA^	99.10 ± 0.43^aA^	99.27 ± 0.73^aA^	99.11 ± 0.34^bA^	100 ± 0.08^aA^	93.11 ± 0.26^bA^	88.66 ± 0.18^bA^	89.87 ± 0.23^bA^	100 ± 1.05^aA^	101.03 ± 0.76^aA^	101.91 ± 1.57^aA^	102 ± 1.77^aA^
Integrity	100 ± 0.59^aA^	98.29 ± 0.69^bA^	98.34 ± 0.58^bA^	98.37 ± 0.58^bA^	100 ± 0.24^aA^	194.59 ± 0.14^bB^	213 ± 0.20^bB^	213.51 ± 0.11^bB^	100 ± 0.70^aA^	101.61 ± 0.76^bA^	102.66 ± 0.82^bA^	104.21 ± 0.69^bA^
PMMA block	100 ± 1.18^aA^	98.76 ± 0.20^bA^	98.83 ± 1.16^bA^	98.73 ± 0.37^bA^	100 ± 0.28^aA^	100 ± 0.05^bA^	120 ± 0.22^bA^	150 ± 0.11^bA^	100 ± 1.43^aA^	98.52 ± 0.19^aA^	100.14 ± 1.39^aA^	100.04 ± 0.30^aA^
Acrylic resin	100 ± 0.48^aA^	99.20 ± 0.31^bA^	99.15 ± 0.37^bA^	99.15 ± 0.29^bA^	100 ± 0.05^aA^	150 ± 0.12^bC^	172 ± 0.11^bB^	175 ± 0.13^bB^	100 ± 0.43^aA^	103.80 ± 0.43^bA^	103.29 ± 0.70^bA^	104.52 ± 0.51^bA^
Coffee												
Flowable	100 ± 1.18^aA^	98.59 ± 0.20^bA^	99.40 ± 1.16^bA^	97.88 ± 0.37^bA^	100 ± 0.28^aA^	303 ± 0.05^bA^	270 ± 0.22^bA^	236 ± 0.11^bA^	100 ± 1.43^aA^	102 ± 0.19^aA^	107 ± 1.39^bA^	103 ± 0.30^aA^
Protemp	100 ± 0.58^aA^	97.96 ± 0.60^bA^	98.08 ± 0.58^bA^	98.39 ± 0.69^bA^	100 ± 0.13^aA^	89 ± 0.17^bB^	91 ± 0.12^bB^	93 ± 0.13^bB^	100 ± 1.24^aA^	100.54 ± 0.26^aA^	106.65 ± 2.41^bA^	100.29 ± 1.80^aA^
Integrity	100 ± 0.39^aA^	96.57 ± 0.35^bA^	96.97 ± 0.94^bA^	97.70 ± 1.41^bA^	100 ± 0.14^aA^	350 ± 0.01^bA^	300 ± 0.29^bA^	280 ± 0.40^bA^	100 ± 1.54^aA^	110.18 ± 0.20^bA^	113.09 ± 1.24^bA^	110.24 ± 1.80^bA^
PMMA block	100 ± 0.12^aA^	98.54 ± 0.05^bA^	98.73 ± 0.13^bA^	98.62 ± 0.09^bA^	100 ± 0.04^aA^	150 ± 0.05^bC^	130 ± 0.06^bB^	102 ± 0.05^bB^	100 ± 0.27^aA^	100.83 ± 0.14^bA^	101.28 ± 0.23^bA^	101.62 ± 0.16^bA^
Acrylic resin	100 ± 0.85^aA^	98.94 ± 0.94^bA^	98.52 ± 0.80^bA^	99.05 ± 0.85^bA^	100 ± 0.10^aA^	175 ±0.09^bC^	187 ± 0.12^bA^	204 ± 0.11^bA^	100 ± 1.04^aA^	104.60 ± 0.81^bA^	106.63 ± 0.88^bA^	105.91 ± 0.76^bA^

*Note*: Lowercase letters indicate significant difference compared to other time points of the same material and beverage within each row.

Uppercase letters indicate significant difference compared to other materials of the same time point, material, and beverage within each column.

Abbreviation: PMMA, polymethyl methacrylate.

The redness of the restoration materials changed to varying degrees in different beverages (Table 3). Flowable resin composite (the control group) displayed no significant change in redness when immersed in orange juice or cola (*p* > .05). The redness of the flowable resin composite significantly decreased (i.e., increased green) on Day 14 when immersed in distilled water (*p* = .03), but it significantly increased in coffee from Day 5 (*p* = .008). The redness of Protemp, too, did not change significantly in orange juice (*p* = .697). Protemp's color was more greenish from Day 5 when immersed in distilled water, cola, or coffee. The behavior of Integrity and PMMA block were similar to each other in terms of redness changes. Their redness significantly increased from day 5 in all beverages (*p* < .05). The redness of acrylic resin significantly increased in distilled water, cola, or coffee from Day 5, but it was more greenish in orange juice from Day 5 (*p* = .015).

The yellowness of the provisional restoration materials changed less than the redness (Table 3). Although there were changes in yellowness in some of the materials in distilled water, none of those changes were significant (*p* > .05). Yellowness increased in all of the provisional restoration materials in all the beverages, except for the Flowable in distilled water and the PMMA block in cola. Yellowness insignificantly decreased in those two cases. In orange juice, the yellowness of all the materials increased significantly on Day 5 (*p* < .05) except for the Protemp, which increased only insignificantly throughout the 14 days in orange juice (*p* = .096). In cola, the yellowness of only Integrity (*p* = .01) and acrylic resin (*p* = .01) significantly increased on Day 5, and increases in all other materials were insignificant. In coffee, the Integrity (*p* < .001), PMMA block (*p* = .039), and acrylic resin (*p* = .004) showed significant increases in yellowness at Day 5. Also in coffee, the yellowness of the flowable resin composite (*p* = .027) and Protemp®4 (*p* = .011) significantly increased at Day 7; however, by Day 14, the yellowness of both of these materials had decreased, with yellowness of these two once again insignificantly different from their initial color.

**Figure 1:**
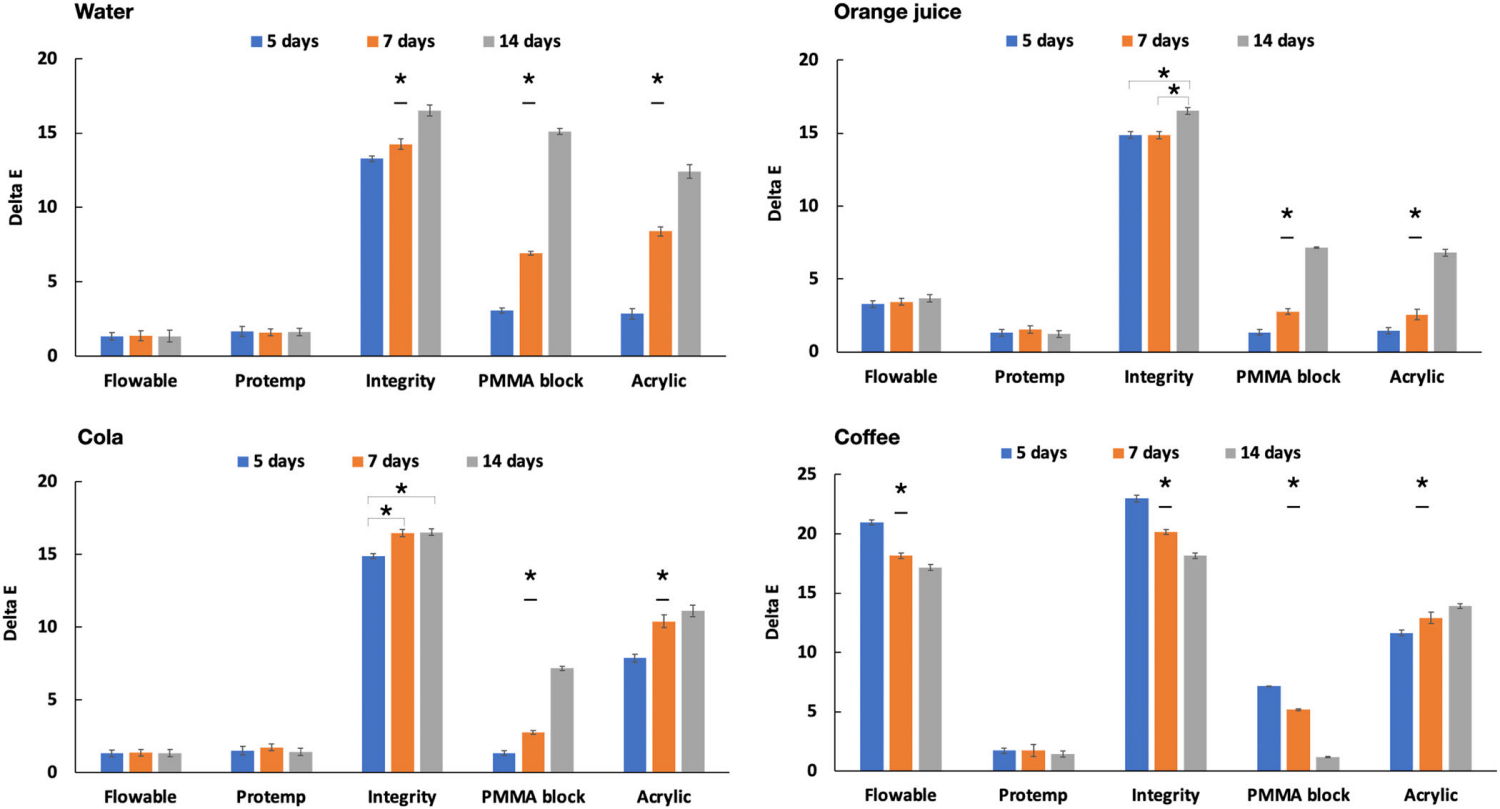
The ΔE of provisional restoration materials in different beverages across 14 days. *All three time periods of this material are significantly different from each other. *Where there is a bracket, the pairs are significantly different.

The color changes of the provisional materials after different periods of time (5, 7, and 14 days) compared with their initial color were measured in ΔE (Figure 1). The Integrity had the highest ΔE in all beverages. The ΔE of the Integrity®, PMMA block, and acrylic resin significantly increased from Day 5 to Day 14 in all beverages except in coffee. In coffee, the ΔE of the Integrity® and PMMA block significantly decreased from Day 5 to Day 14. The flowable resin composite had a ΔE around 1.5 or 4 in distilled water, orange juice, and cola, and there was no significant difference in ΔE throughout the 14 days (*p* > .05). The ΔE of the flowable resin composite reached its peak of 20.97 in coffee on Day 5, before significantly decreasing to 18.15 and 17.15 on Days 7 and 14, respectively (*p* < .05). The ΔE of the Protemp hovered around 1.5–2 for 14 days in all the beverages, and there was no significant difference in ΔE among different time periods (*p* > .05).

### 3.2 The hardness of the provisional restoration materials in different beverages

**Figure 2:**
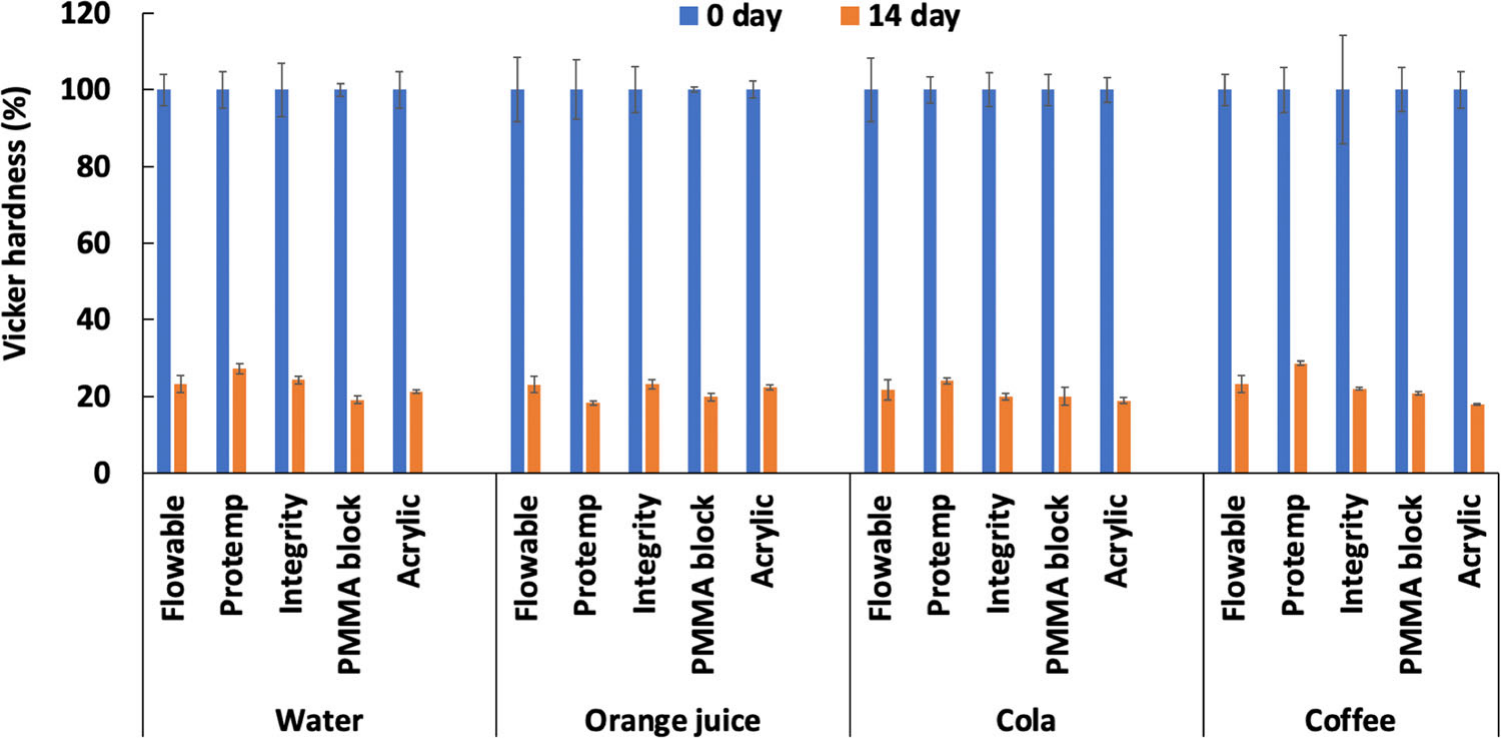
The hardness of provisional restoration materials in different beverages at Day 0 and Day 14.

The hardness of the provisional materials in different beverages was tested after 14 days, and the results are shown in Figure 2. All the provisional material types significantly decreased in hardness compared to their initial hardness (*p* = .012). There was no statistical difference between materials after being submerged for 14 days in the beverages: water (*p* = .113), orange juice (*p* = .717), cola (*p* = .275), and coffee (*p* = .149).

### 3.3 The surface roughness of the provisional restoration materials under different beverages

**Figure 3:**
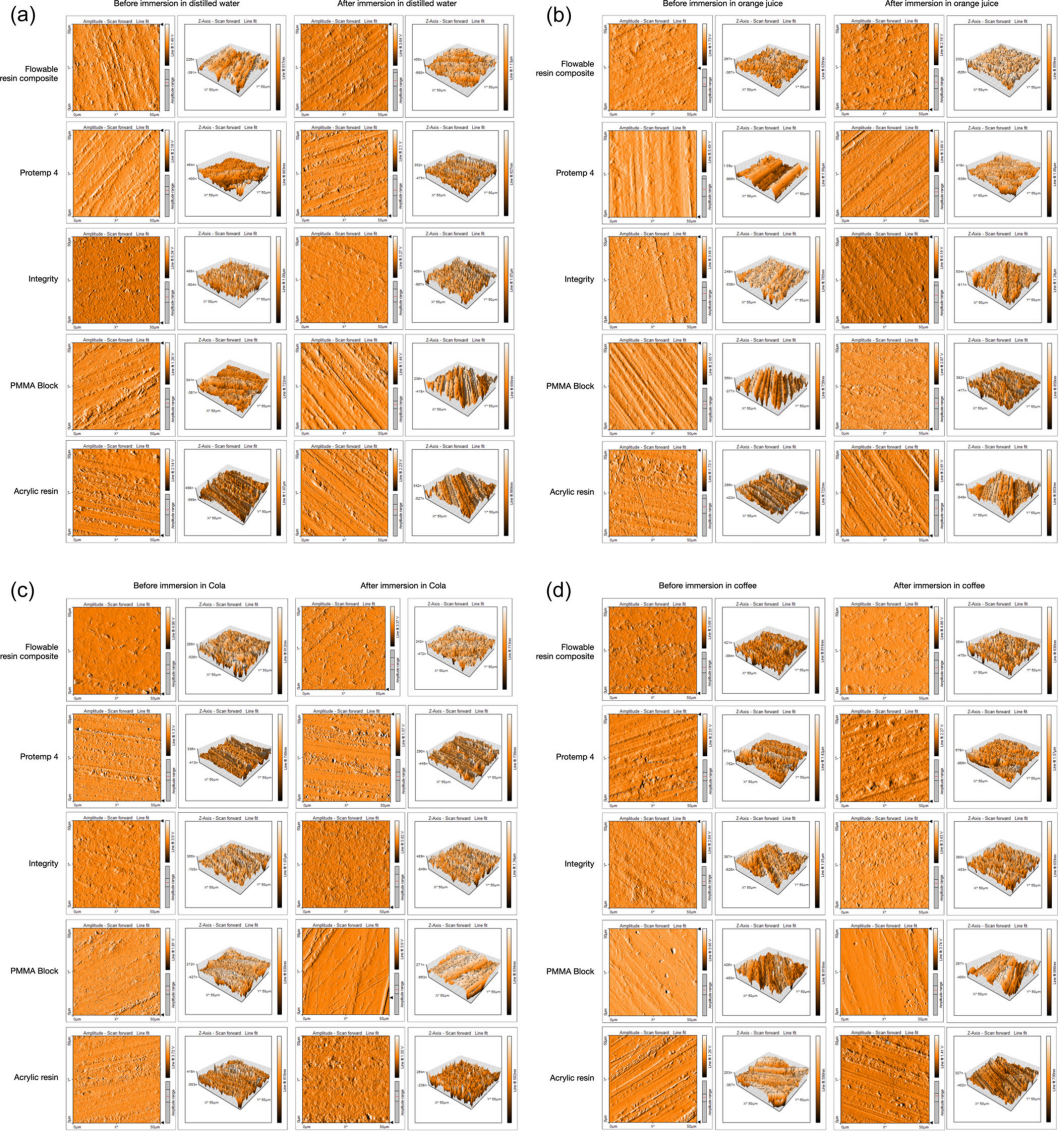
Surface roughness profiles of the provisional restoration materials before and after immersion in distilled water (a), orange juice (b), cola (c), and coffee (d).

The surface roughness of the provisional restoration materials showed no change when immersed in distilled water for 14 days. However, in orange juice, the roughness of the PMMA block significantly decreased (*p* = .022) compared to its initial measurement. In contrast, the Integrity and Acrylic in Cola significantly increased (*p* = .008) compared to their initial measurements. The surface roughness of the provisional restorative materials was detected using the AFM, and the resulting three‐dimensional figures can be seen in Figure 3.

After the different resin‐based materials were immersed in the different beverages, Protemp®4 had the lowest color change of all beverages. Flowable resin composite and Protemp®4 had the lowest changes in surface roughness. All the materials decreased in their hardness with no significant difference between materials in the amount of the decrease.

## 4 DISCUSSION

The provisional restoration materials in this study can be divided into two groups according to their principle monomer: the methyl methacrylate group and the dimethacrylate group. The PMMA block and acrylic resin make up the methyl methacrylate group, whereas the Protemp®4 and Integrity® are the dimethacrylate group. The latter, which are bis‐acrylic composites (also known as bis‐acrylate composites), share some similarities with resin composite materials, namely their monomer composition and inorganic fillers (Kerby et al., 2013; Vaidyanathan et al., 2015). The dimethacrylate monomer has higher cross‐linkage than the methyl methacrylate monomer, leading to lower water sorption and a lower diffusion coefficient (Smith et al., 2015). Consequently, the dimethacrylate group has less polymerization shrinkage and exothermal expansion compared to the methyl methacrylate group (Kerby et al., 2013; Vaidyanathan et al., 2015).

The discoloration of resin‐based materials commonly occurs in the resin matrix, and it can be associated with intrinsic or extrinsic factors. Intrinsic factors are related to the dental materials themselves, for example, their chemical composition, their types of photoinitiators, and their types of polymerizations (Schneider et al., 2008). Some examples of common extrinsic factors are food, drinks, and tobacco.

In the case of internal discoloration, the color can change immediately during initial polymerization, because when photoinitiators such as camphorquinone are activated by light, their chromophores break, resulting in a diminished b* value (Asmusen et al., 2009; del Mar Pérez et al., 2009). This process continues even after completion of the light‐curing procedure, during postirradiation polymerization (also known as dark curing). Although distilled water is colorless, the color of dental materials commonly changes when immersed in distilled water for a period of time due to the chemical composition in the polymer network. This is also internal discoloration. In this study, when all the materials were immersed in distilled water, flowable resin composite was the only material that decreased in b* during the 14 days. The decrease was not statistically significant. The other materials, all of which are chemical cured, increased in b*. The self‐cured resin‐based materials use benzoyl peroxide and tertiary amine to initiate polymerization. The degradation of tertiary amine and oxidation of unreacted carbon–carbon double bonds cause discoloration (Bogis & Turgut, 2011). When immersed in distilled water, three of the four chemical‐cured provisional restoration materials (Integrity®, PMMA block, and acrylic resin) significantly increased in ΔE throughout the 14 days. Only Protemp®4, which is also chemical cured, insignificantly changed in color. This result is consistent with a previous study (Cunha et al., 2017). The Protemp®4 has a higher filler content than the Integrity®, which is also a bis‐acrylic composite. That explains the Protemp®4 having lower water absorption, resulting in less discoloration (Mei et al., 2015).

In this study, examples of external discoloration can be seen from the colored beverages. The color of orange juice comes from carotenoids, natural pigments found in plants and animals. Carotenoids are hydrophobic and lipophilic compounds. They are soluble in alcohol, acetone, or chloroform (Rivera & Canela, 2012). Cola is made by dissolving carbon dioxide into water for carbonation and adding sugar, flavors, and coloring. Cola gets its brown color from caramel color, which is made by heating sugar or other carbohydrates (Smith et al., 2015). Cola also contains caffeine, but caffeine is not a chromogen, so it is not responsible for staining. However, the acidity of cola can erode the surfaces of substrates, making it easier for the caramel color in cola to cause stains (Pirolo et al., 2014). The discoloration of substances by coffee is associated with tannins and chlorogenic acid in the coffee. Tannins are phenolic compounds found in plants. They are soluble in water, alcohol, sodium hydroxide, or acetone, with the highest solubility in water (Antwi‐Boasiako & Animapauh, 2012). In the process of extracting tannins with water, sugar, and gum are also commonly obtained, and these can make tannins stickier, which makes them useful as an adhesive in the wood industry (Das et al., 2020). Tannins are able to bind to proteins, including the saliva proteins, particularly the proline‐rich proteins. Proline‐rich proteins are also a component of tooth pellicle (Adamczyk et al., 2017; Gupta & Gupta, 2011). Therefore, brown coffee stains commonly discolor tooth surfaces.

Flowable resin composite generally behaves differently in the discoloration results than the other materials in this study. The flowable had the lowest ΔE of all materials when immersed in distilled water and cola, and it had the second lowest ΔE in orange juice. This good performance can be explained by the monomer in flowable. Flowable resin composite is the only material in this study that uses Bis‐GMA. Bis‐GMA is a dimethacrylate monomer that shows lower water sorption than other dimethacrylate monomers because it has intramolecular hydrogen bonds. These bonds make the structure denser and tighter (Lemon et al., 2007). However, when immersed in coffee, flowable resin composite was nearly the most discolored material, surpassed only slightly by Integrity. Another point that stands out is that although the flowable ΔE in water, cola, and orange juice increased (albeit insignificantly) over time, it continuously decreased significantly in coffee. These results suggest that the mechanism by which the various beverages discolor the materials differs, particularly in the case of coffee. The tannic acid in roasted coffee beans is the main cause of coffee stains. The pH of this acid is 5.13, which is lower than the critical pH of enamel. That causes demineralization of the enamel and easy deposition of chromogenic agents (Gupta & Gupta, 2011). This suggests that the staining procedure of coffee involves chromogen deposition on the rough surface of the materials or adherence to the pellicle protein of the tooth rather than diffusion into the substrates. Previously, Omata et al. also found that the staining behavior of resin composite in coffee was different from other beverages such as tea or red wine when immersion lasted 4 weeks. In that study, after the first week in coffee, the color of the resin composite remained unchanged for the remaining 3 weeks. In contrast, when the resin composite was immersed in tea or red wine, discoloration continuously increased throughout 4 weeks (Omata et al., 2006). Not only the resin composite but actually the majority of materials in coffee gradually decreased in ΔE after the initial stain. One possible explanation is that the particular chromogens in question might be vulnerable to being partially washed away when the materials are rinsed during the periodic refreshing of each beverage solution.

Absorption and adsorption of colorants in the beverages cause discoloration of the provisional materials. The resin‐based materials have numerous fine pores generated during polymerization and subsequent use (Nilsen et al., 2020). The available surface and pore volume of materials together with the chemical properties of the beverages directly affect the amount of adsorption by the materials. Daily brushing with dentifrice is able to remove only adsorbed stains. However, the discoloration of resin‐based materials decreases only insignificantly after brushing with dentifrice, particularly coffee stains (Zimmerli et al., 2012). Even though this study evaluated the discoloration of materials without mimicking brushing, the study can demonstrate the effects of beverage colorant staining on provisional materials.

Previous studies found that the highest discoloration of resin‐based materials occurred in coffee (Sulaiman et al., 2020). Nanofilled resin composite had the least discoloration in color beverages such as coffee, tea, and cola (Sulaiman et al., 2020), but it had high discoloration in fruit/vegetable/herbal beverages, particularly in grape juice and turmeric solution (Fontes et al., 2009). Similar to the current study, all the types of provisional materials, except for Protemp, had more discoloration in coffee, and the discoloration of the nanofilled flowable resin composite markedly increased in orange juice and coffee. Among the provisional restorative materials, some studies showed that PMMA block had the lowest discoloration, followed by heat‐cured PMMA, self‐cured PMMA, and Bis‐acryl resin composite (Mazaro et al., 2015). Those results were generally similar to the current study in which the PMMA block had the second‐lowest discoloration, followed by acrylic resin and Integrity. The current study found that Protemp had the lowest discoloration, which was different from previous studies. This difference might be related to the incubating process. In the current study, all samples were mechanically agitated throughout the incubation period to prevent any possible settling of the solutions, while almost all of the previous studies were performed without any agitation during incubation. This might explain the different results in material discoloration.

A spectrophotometer is an analytical instrument used to quantitatively measure the reflection or absorption properties of a material (Karaagaclioglu et al., 2010). The spectrophotometer disperses only light‐emitting diode (LED) light through the material and interprets only the results of LED light, so this interpretation is not affected by ambient conditions, as long as there is no other source of LED light in the room. Wavelengths from the spectrophotometer that are reflected back from the material are analyzed and interpreted by the machine to match the color shades of the VITA shade standards.

The hardness of all the provisional restoration materials significantly decreased when immersed in any of the beverages, including distilled water. Previous studies have shown that the surface of resin‐based materials becomes distinctly softened when immersed in distilled water for 6 months (Martos et al., 2003). Water uptake is the main factor influencing the various properties of materials. When resin‐based materials are immersed in water, the resin matrix swells, leading to a reduction of the friction forces between polymer chains. This is because water functions as a plasticizer within the resin matrix (Alrahlah et al., 2022). Even though the surfaces of the samples were covered using a glass slide during polymerization, the oxygen inhibition layer still occurred to some extent. This is why both sides of all samples in this study were polished with sandpaper to remove the unpolymerized resin. In addition, the hardness of subsurface areas is significantly higher than the top layers. This is related to the polymerization procedure of materials. During polymerization, the lower layers of a material rise to a higher temperature than the top layer (Asmussen & Peutzfeldt, 2003). Even though the difference in temperature may not be great, it is still enough to significantly increase hardness below the top layer (Taubock et al., 2011).

Surface roughness or surface irregularity is evaluated by the deviations in the direction of the normal vector of a real surface from its ideal form. The common parameter to define surface roughness is arithmetical mean roughness (Ra), which is determined as a line on the surface of the restorations (Yahya et al., 2020). Instead of using a line, this study determined the roughness of the restoration surface by examining a sample area, because this method provides more information about the material. The roughness of the dental restoration surfaces results in plaque and stain retention, gingival inflammation, and solubility of the components (particularly the organic matrix, due to acid from plaque) (Petropoulou et al., 2020; Rashid, 2014; Roeder et al., 2000). Surface roughness higher than Ra 0.2 μm facilitates biofilm formation (Yu et al., 2016), including biofilm populations that lead to periodontal disease. The roughness also influences the esthetics of the materials as well as the wear of the restoration itself or the antagonistic surfaces (Ghazal & Kern, 2009; Habib et al., 2020). In resin‐based materials, the cohesion of the organic matrix and the shape and amount of the filler particles have an influence on polishable surfaces. High surface roughness usually indicates that filler particles are protruding, and this is the main cause of abrasion with the antagonist enamel (Branco et al., 2020). In this study, some beverages increased the surface roughness of the materials, and this might accelerate the wear of the antagonist enamel beyond just the direct acidic effect of the beverages on the tooth enamel.

The surface roughness of the provisional restoration materials did not change notably in most of the experimental groups. Exceptions were the surfaces of Integrity® and acrylic, which significantly increased in roughness after immersion in cola for 14 days. Phosphoric acid is a common component in colas (Tahmassebi et al., 2006). It softens the resin matrix of polymers (Tap et al., 2000). Resin‐based materials usually absorb water, and water promotes the penetration of acid into the resin matrix (Bagheri et al., 2005; Schulze et al., 2003). Filler particles cannot absorb water in their bulk, but they can absorb water on their surface. When resin‐based materials contain abundant water and acid, this will accelerate the expansion of the resin matrix and hydrolyzation of silane, leading to microcracks in the resin matrix and interfacial gaps between filler particles and the resin matrix (Bagheri et al., 2005). The acidity of cola therefore roughens the material surfaces and accelerates both decomposition in the resin matrix and detachment of the filler particles of resin‐based materials (Schulze et al., 2003). Multiple previous studies have found that carbonated beverages contain added acidity regulators (phosphoric acid, malic acid, and citric acid) (Pinto et al., 2013) that cause hydrolysis of ester radicals in resin monomers and lead to degradation of resin composite materials (Ayre et al., 2014; Paula Mathias, 2014).

Although the four provisional restorative materials in this study intentionally span a variety of monomer types and manufacturing techniques, statistically there are insufficient representatives of each monomer type and manufacturing technique for the purposes of analyzing the specific influence of these two factors on the tolerance of the materials to daily beverages. Rather, the present report evaluated the color, hardness, and roughness of the four specific materials used. It would also be interesting in the future to observe either microstructure and/or chemical composition (Tseng et al., 2021) after these materials have performed under different beverages.

## 5 CONCLUSION

The composition of resin‐based materials is a crucial factor influencing their absorption of liquids. The surface characteristics and durability of these materials also depend on the penetration of liquid. The types of chromogens present in a food or beverage affect the depth and persistence of the stains it produces on resin‐based materials.

## AUTHOR CONTRIBUTIONS

**Kornchanok Wayakanon** conceived the study, designed the protocol, contributed to data collection and preparation, wrote the manuscript, and contributed to the interpretation of the results. **Teeraphan Narakaew** was responsible for data analysis. **Praween Wayakanon and Kornchanok Wayakanon** were responsible for critical revision of the article for important intellectual content and approval of the final version.

## CONFLICT OF INTEREST STATEMENT

The authors declare no conflicts of interest.

## Data Availability

Raw data can be requested through the corresponding author.
